# Adapting to Health Change: Aging Bias, Sensitivity, and Interprofessionalism in an Aging Sensitivity Training

**DOI:** 10.1093/geroni/igaa057.3259

**Published:** 2020-12-16

**Authors:** Keith Kleszynski, Lee Jennings, Thomas Teasdale, Carrie Ciro, Carol Rogers, Tina Peterson, Keith Swanson, Beavers Kay

**Affiliations:** 1 University of Oklahoma Health Sciences Center, Oklahoma City, Oklahoma, United States; 2 University of Oklahoma Health Sciences Hudson College of Public Health, Oklahoma City, Oklahoma, United States; 3 University of OKlahoma Health Sciences Center, Oklahoma City, Oklahoma, United States; 4 University of Oklahoma Health Sciences Center, Reynolds Center for Geriatric Nursing Excellence, Oklahoma City, Oklahoma, United States; 5 University of Oklahoma, Norman, Oklahoma, United States

## Abstract

Adapting to Health Change, a 2-hour aging sensitivity simulation to increase student sensitivity to age-related changes, was delivered to 148 health professions students in March 2020. Five small group stations (hearing loss, neuropathy and dexterity, vision loss and medication management, language and cognitive impairment, and mobility and balance) were facilitated by older adult volunteers and interdisciplinary faculty. Students completed (pre-post) Aging IQ Quiz, Aging Attitudes Assessment, Inter-professional Attitudes Scale (IPAS) Teamwork, Roles, and Responsibilities and Interprofessional Biases subscales, and an overall satisfaction rating. There was a statistically significant but small change in perceived knowledge of aging (mean Aging IQ score changed -0.025 points, p = 0.03). We also found a post-training increase in negative bias (mean Aging Attitudes Assessment score changed +2.68 points, p = 0.0001). While there was no change in attitudes about interprofessional education, this wasattributed to high baseline scores, suggesting students already valued interprofessional interactions. Multiple high satisfaction scores were encouraging. Ninety-four percent agreed that the simulation increased their understanding of age-related changes, while 97% indicated facilitators provided useful insights into the experiences of aging. A high majority (89%) felt they would be better health care providers for older patients than they would have been without the experience. This exercise to increase student empathy about age-related disabilities was well received, but did not achieve the usual intended aims. Simulation content should “re-aim” beyond empathy to teach new knowledge, highlight positive aspects of aging and professional care provision, and reinforce interprofessional roles toward wellness for older adults.

